# A Time-of-Flight and Radar Dataset of a neonatal Thorax Simulator with synchronized Reference Sensor Signals for respiratory Rate Detection

**DOI:** 10.1038/s41597-024-02946-5

**Published:** 2024-01-23

**Authors:** Johanna Gleichauf, Sven Herrmann, Christine Niebler, Alexander Koelpin

**Affiliations:** 1 Nuremberg Institute of Technology, Department of Electrical Engineering, Precision Engineering and Information Technology, Nuremberg, 90489 Germany; 2 Hamburg University of Technology, Institute of High-Frequency Technology, Hamburg, 21073 Germany

**Keywords:** Neonatology, Preterm birth

## Abstract

In this paper we present an open-source Time-of-Flight and radar dataset of a neonatal thorax simulator for the development of respiratory rate detection algorithms. As it is very difficult to gain recordings of (preterm) neonates and there is hardly any open-source data available, we built our own neonatal thorax simulator which simulates the movement of the thorax due to respiration. We recorded Time-of-Flight (ToF) and radar data at different respiratory rates in a range of 5 to 80 breaths per minute (BPM) and with varying upstroke heights. As gold standard a laser micrometer was used. The open-source data can be used to test new algorithms for non-contact respiratory rate detection.

## Background & Summary

Preterm neonates require continuous monitoring of their vital parameters. The current monitoring methods (e.g. ECG, pulse oximetry, etc.) have in common that they require direct contact to the neonate′s sensitive skin. This can lead to skin irritations, pressure marks, eczema and even the removal of the skin. To reduce these risks non-contact monitoring methods are being developed. To develop such algorithms data for testing and validation is required. To gain real data is expensive and difficult due to ethical and regulatory restrictions. There exist no open-source datasets as this data is medical patient data. In order to develop non-contact respiratory rate detection algorithms based on ToF and radar data, a neonatal thorax simulator was designed in previous work which simulates the thorax movement due to respiration^1^. We recorded datasets of 15 minutes at different respiratory rates (5, 10, 20, 30, 40, 45, 50, 55, 60 and 80 BPM) and in different modes (*normal* and *deep*).

## Methods

**Fig. 1:**
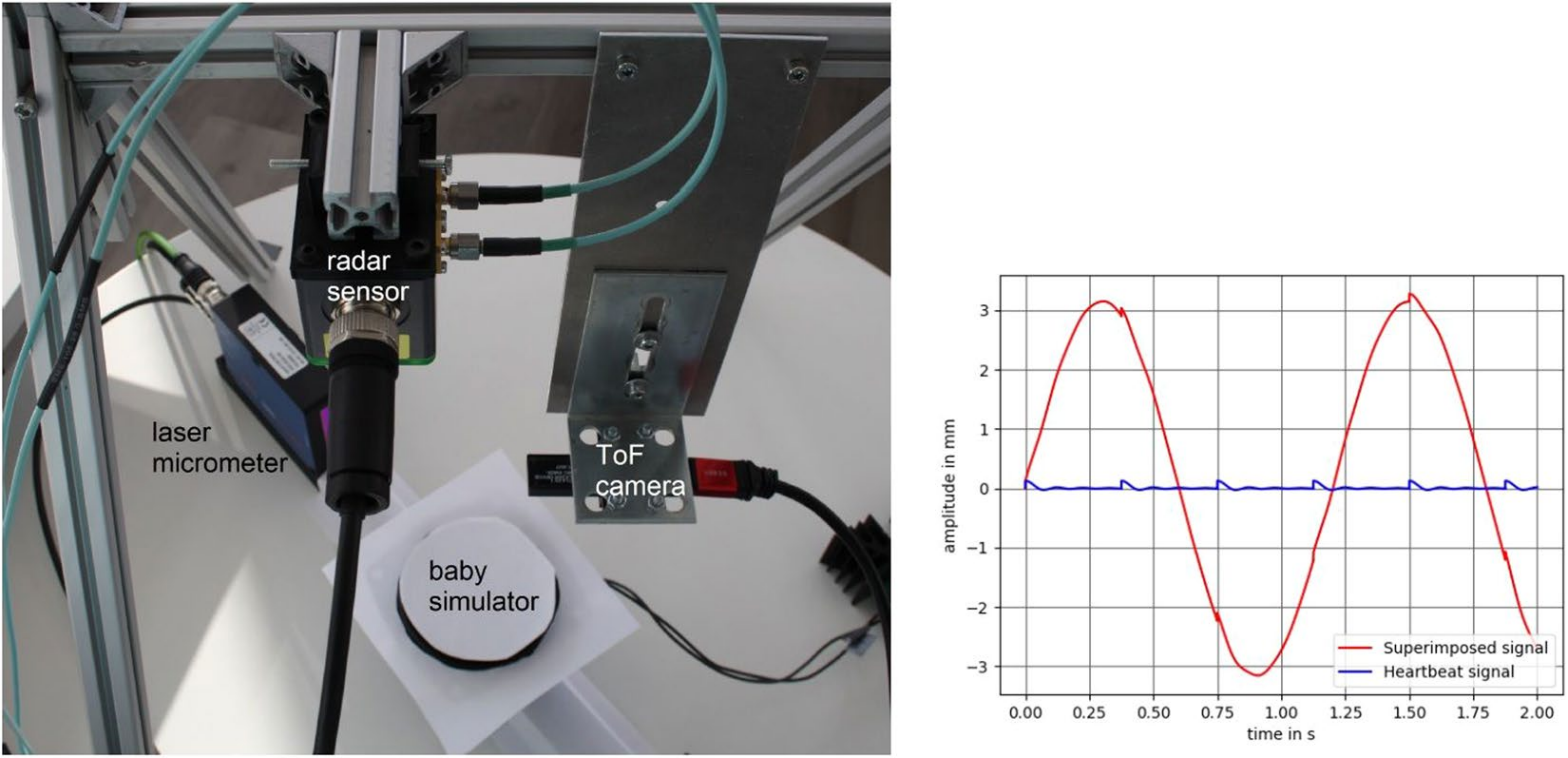
Hardware setup for the ToF-radar system (left). The neonatal thorax simulator can be seen in the middle, and the laser micrometer serves as reference measurement^1^. On the right the simulated signal can be seen.

In this section we describe the concept behind our neonatal thorax simulator as well as all used sensors for the recording of this dataset. The recordings were part of our previous work^1^. The recording setup is shown in Fig. 1. We will now describe each part in detail.

### Neonatal thorax simulator

Our neonatal thorax simulator simulates the thorax movement due to respiration and is made up of a speaker (by company Visaton (https://www.conrad.de/de/p/visaton-fr-10-4-zoll-10-16-cm- breitband-lautsprecher-chassis-20-w-4-303634.html)), an amplifier circuit (by company Conrad (https://www.conrad.de/de/p/conrad-components-stereo-verstaerker-bausatz-9-v-dc-12-v-dc-18-v-dc-35-w-2-1216582.html)) and a computer. The generation of the respiratory signals is done by a software program on the computer. Our idea was to superimpose two sine waves: one for the respiration and a decaying sine for the heartbeat for future developments. We are aware that real breathing patterns are more complex than a sinusoidal signal which would require the time for breathing in and out as well as holding the breath to be set. Unfortunately, the amplifier used for our system is incapable of passing low frequency signals, which would be necessary for generating the plateau part of the pattern. Futhermore, the speaker would not cope with the DC-signals. Therefore, we use the simplified sine signal as for now.

The resulting signal (see Fig. 1) is then transmitted from the AUX output of the computer to the speaker via the amplifier circuit. Due to the small output power of the computer, the amplifier was required to reach the desired amplitude in the speakers membrane displacement. The electrical signal is then transformed into the movement of the speaker membrane.

The simulator has two respiratory simulation modes: *normal* and *deep*, with *deep* mode providing strokes of twice the size than *normal* mode. The exact stroke sizes can be found in Table 1.

**Table 1:** Measured strokes with the laser micrometer in *deep* and *normal* mode^1^.

BPM	Interbeat interval	Stroke in mm	
		*deep* mode	*normal* mode
5	12 s	0.305	0.136
10	6 s	0.644	0.273
20	3 s	1.344	0.589
30	2 s	1.965	0.928
40	1.5 s	2.591	1.285
45	1.33 s	2.797	1.394
50	1.2 s	3.047	1.66
55	1.09 s	3.285	1.72
60	1 s	3.472	1.917
80	0.75 s	4.068	2.481

The code for generating the signals to be simulated by the speaker can be found within our figshare repository^2^.

### Time-of-Flight camera

The 3D Time-of-Flight camera uses the phase difference method for calculating the distance to objects (https://pmdtec.com/picofamily/faq/). The distance is determined using the phase shift *ϕ* and the wavelength *λ* of the reflected modulated signal:

1 $$d=\frac{\phi }{2\cdot \pi }\cdot \frac{\lambda }{2}$$

The 3D imager detects the infrared light reflection and then uses the phase shift for calculating the distance to the object. This results in a 3D point cloud.

**Fig. 2:**
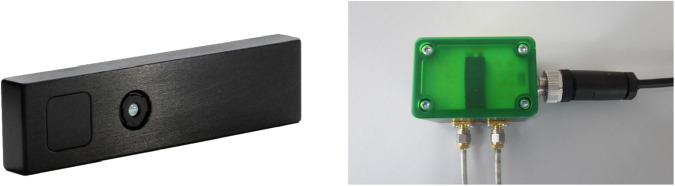
Pico flexx 3D time-of-flight camera by pmd (https://pmdtec.com/picofamily/wp-content/uploads/2018/03/PMD_DevKit_Brief_CB_pico_flexx_CE_V0218-1.pdf) (left) and radar sensor by InnoSenT (right)^1^.

We used the CamBoard pico flexx 3D time-of-flight camera by the company pmd (see Fig. 2). The measurement range lies between 0.1–4m. The camera has a field of view (FoV) of 62° × 45° and a resolution of 224 × 171 (38k) pixels. A frame rate of up to 45 fps (3D frames) can be selected. For using the camera an Ubuntu 16.04 computer with Robot Operating System (ROS) Kinetic installed is required. The driver used is the Royale SDK provided by pmd as well as the ROS package *pico_flexx_driver* (https://github.com/code-iai/pico_flexx_driver). We set the frame rate to 35 fps as this is a frame rate commonly used for cameras in this context.

### Radar sensor

The radar sensor we used is a microwave interferometric radar sensor based on Continuous Wave (CW) radar. One characteristic of this sensor is that no Fourier transform is needed and only the phase shift between the reflected and the reference microwave has to be determined. The relative distance is calculated using the following formula:

2 $$\Delta x=\frac{\Delta \sigma }{2\cdot \pi }\cdot \frac{\lambda }{2}$$

with Δ*x* as relative distance, Δ*σ* as relative phase shift and *λ* as wavelength. The relative phase shift can also be represented by its In-Phase (I) and Quadrature (Q) signal:

3 $$\Delta \sigma =\arctan \frac{I}{Q}$$

We chose the microwave interferometric radar sensor iSYS-4001 by the company InnoSenT (see Fig. 2). The radar sensor was originally designed for the research project GUARDIAN (https://www.technik-zum-menschen-bringen.de/projekte/guardian), which is applied for the monitoring of vital parameters of patients in the palliative care unit. The frequency of the radar sensor lies at 24.2 GHz and it has a power of 100 mW (20dBm) which lies within the legally approved norm range. An Infineon XMC4500 microcontroller receives and transfers the radar signal. The raw data (In-Phase (I) and Quadrature (Q) signal) is digitized by an analog-to-digital converter (ADC), ADS1298 by Texas Instruments, due to its higher performance than the inbuilt ADC of the Infineon XMC4500 microcontroller. This is done with a sampling rate of 2 kHz. The digitized data is then sent via Ethernet to the computer.

### Reference laser micrometer

**Fig. 3:**
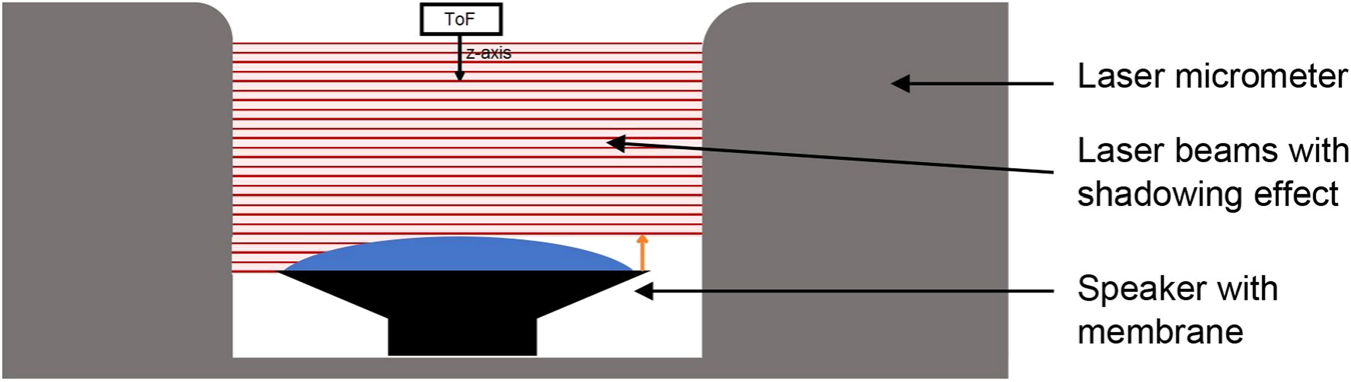
Hardware setup of the neonatal thorax simulator. The speaker membrane changes its upstroke size. We are only interested in the change of height which is detected by the light curtain (see orange arrow)^1^.

As gold standard a precise reference measurement has to be used. This sensor needs to have the following characteristics: it has to be able to detect small movements such as the displacement of the thorax even due to the heartbeat (for future work), which lie within the submillimeter range. A laser micrometer based on the principle of shadowing is a good choice (https://www.micro-epsilon.co.uk/service/glossar/optoCONTROL.html, https://www.micro-epsilon.co.uk/download/manuals/man–optoCONTROL-2520–en.pdf). When an object moves into the light carpet, a change can be sensed (see Fig. 3).

We used the optoCONTROL 2520 laser micrometer by MICRO-EPSILON (https://www.micro-epsilon.co.uk/2D_3D/optical-micrometer/micrometer/optoCONTROL_2520/). The maximum resolution is 1µm and has a measurement range of 46 mm. The inbuilt laser is of laser class 1M, such that no harm is applied to the user. But it should not be magnified by any optical instrument. The laser micrometer’s receiver and transmitter are connected via a CE2520-1 cable. A 24 V power supply is needed. For starting the measurement the optoCONTROL is connected to a Microsoft Windows 10 laptop via Ethernet. The manufacturer delivers a measurement tool called ODC2520 DAQ tool, which was installed. The tool automatically searches for a laser micrometer and connects to it. For our purpose, the mode “Edge light-dark” needs to be selected within its web interface.

## Data Records

Our herein published dataset contains radar, ToF, and laser micrometer reference data. The data was recorded for the following respiratory rates within *deep* and *normal* mode: 5, 10, 20, 30, 40, 45, 50, 55, 60 and 80 BPM. For the radar sensor the IQ values are saved within a csv file with the corresponding timestamps. The ToF pointcloud measurements were saved as png images such that the z-value was saved at the correct coordinate value of the image. The timestamps are found within the png image name. The laser micrometer delivers height values which are saved with the corresponding timestamps within csv files. An overview over all files can be found within Tables 2 and 3. For those interested in our algorithm results, please refer to our results within our previous work^1^.

**Table 2:** Overview datasets 5–40 BPM.

Name	BPM	Mode	Modality	Filename
05BPM_normal	5	normal	radar	iq_values_05BPM_normal.csv
			ToF	ToF_05BPM_normal
			laser	laser_05BPM_normal.csv
05BPM_deep	5	deep	radar	iq_values_05BPM_deep.csv
			ToF	ToF_05BPM_deep
			laser	laser_05BPM_deep.csv
10BPM_normal	10	normal	radar	iq_values_10BPM_normal.csv
			ToF	ToF_10BPM_normal
			laser	laser_10BPM_normal.csv
10BPM_deep	10	deep	radar	iq_values_10BPM_deep.csv
			ToF	ToF_10BPM_deep
			laser	laser_10BPM_deep.csv
20BPM_normal	20	normal	radar	iq_values_20BPM_normal.csv
			ToF	ToF_20BPM_normal
			laser	laser_20BPM_normal.csv
20BPM_deep	20	deep	radar	iq_values_20BPM_deep.csv
			ToF	ToF_20BPM_deep
			laser	laser_20BPM_deep.csv
30BPM_normal	30	normal	radar	iq_values_30BPM_normal.csv
			ToF	ToF_30BPM_normal
			laser	laser_30BPM_normal.csv
30BPM_deep	30	deep	radar	iq_values_30BPM_deep.csv
			ToF	ToF_30BPM_deep
			laser	laser_30BPM_deep.csv
40BPM_normal	40	normal	radar	iq_values_40BPM_normal.csv
			ToF	ToF_40BPM_normal
			laser	laser_40BPM_normal.csv
40BPM_deep	40	deep	radar	iq_values_40BPM_deep.csv
			ToF	ToF_40BPM_deep
			laser	laser_40BPM_deep.csv

**Table 3:** Overview datasets 45–80 BPM.

Name	BPM	Mode	Modality	Filename
45BPM_normal	45	normal	radar	iq_values_45BPM_normal.csv
			ToF	ToF_45BPM_normal
			laser	laser_45BPM_normal.csv
45BPM_deep	45	deep	radar	iq_values_45BPM_deep.csv
			ToF	ToF_45BPM_deep
			laser	laser_45BPM_deep.csv
50BPM_normal	50	normal	radar	iq_values_50BPM_normal.csv
			ToF	ToF_50BPM_normal
			laser	laser_50BPM_normal.csv
50BPM_deep	50	deep	radar	iq_values_50BPM_deep.csv
			ToF	ToF_50BPM_deep
			laser	laser_50BPM_deep.csv
55BPM_normal	55	normal	radar	iq_values_55BPM_normal.csv
			ToF	ToF_55BPM_normal
			laser	laser_55BPM_normal.csv
55BPM_deep	55	deep	radar	iq_values_55BPM_deep.csv
			ToF	ToF_55BPM_deep
			laser	laser_55BPM_deep.csv
60BPM_normal	60	normal	radar	iq_values_60BPM_normal.csv
			ToF	ToF_60BPM_normal
			laser	laser_60BPM_normal.csv
60BPM_deep	60	deep	radar	iq_values_60BPM_deep.csv
			ToF	ToF_60BPM_deep
			laser	laser_60BPM_deep.csv
80BPM_normal	80	normal	radar	iq_values_80BPM_normal.csv
			ToF	ToF_80BPM_normal
			laser	laser_80BPM_normal.csv
80BPM_deep	80	deep	radar	iq_values_80BPM_deep.csv
			ToF	ToF_80BPM_deep
			laser	laser_80BPM_deep.csv

The dataset is available within the following figshare repository^2^.

## Technical Validation

**Fig. 4:**
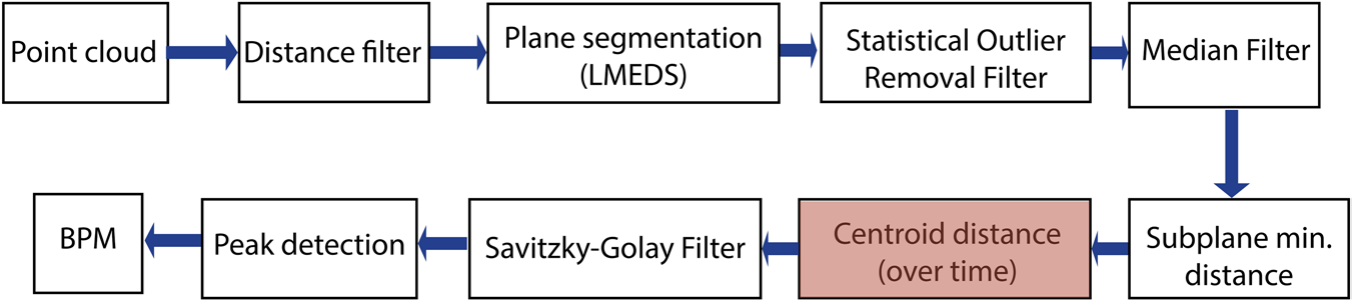
ToF signal processing flow chart to generate the distance signal^1^.

**Fig. 5:**
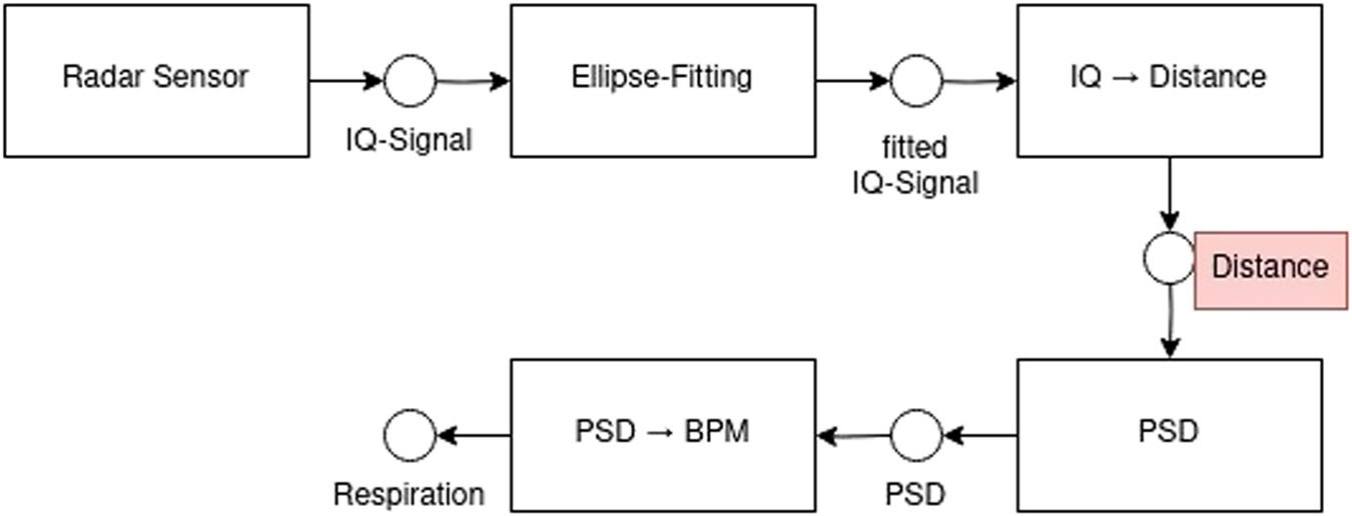
Radar signal processing flow chart to generate the distance signal.

In order to ensure a high quality dataset, first of all the correctness of the laser micrometer measurements had to be confirmed. The set BPM from the simulator were compared with the BPM measured with the laser micrometer by manually counting the peaks. Following, the laser micrometer was used as reference for the ToF camera and radar data. Before comparing the distance measurements several signal processing steps had to take place to receive the distance signal from the camera and the radar sensor. These can be found in Figs 4 and 5 displaying the flowchart. The marked component shows the distance we are using for comparison.

**Fig. 6:**
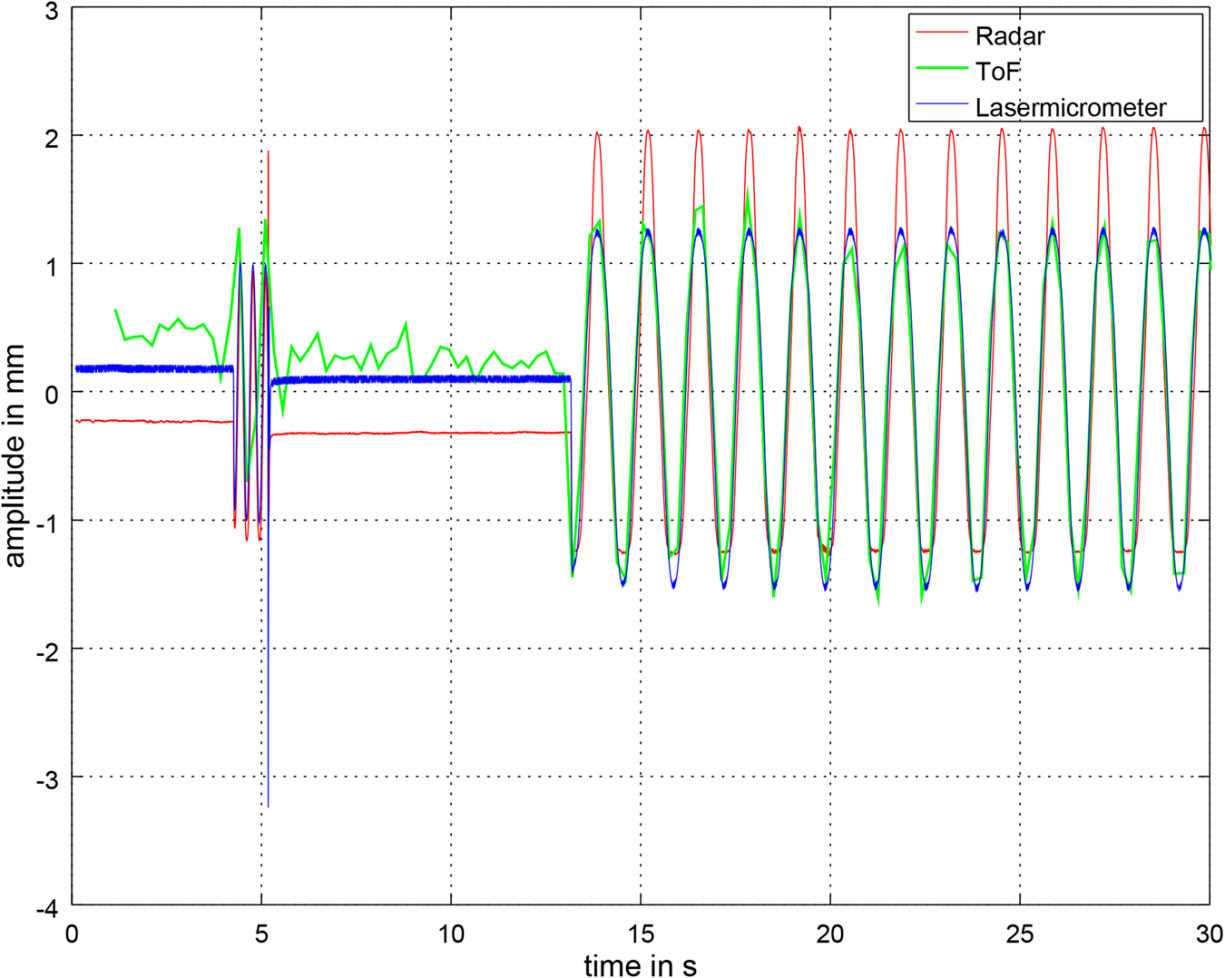
The figure shows the synchronized distance signals at 45 BPM. At the beginning a synchronization signal can be observed. All signals are in sync. The amplitude of the ToF signal is very similar to the laser micrometer signal. The radar amplitude differs due to Ellipse fitting. Further information on this phenomenon can be found in^1^.

Figure 6 shows the synchronized distance signals at 45 BPM. At the beginning a synchronization signal can be observed. All signals are in sync. The amplitude of the ToF signal is very similar to the laser micrometer signal. The radar amplitude differs due to Ellipse fitting. Further information on this phenomenon can be found in^1^.

As the time domain signals in Fig. 6 show a good correspondence, we believe the same will be given in case of a more realistic non-sinusoidal signal with plateaus, as the here used DC-coupled interferometric radar sensor as well as the ToF camera are very sensitive in measuring static positions.

**Fig. 7:**
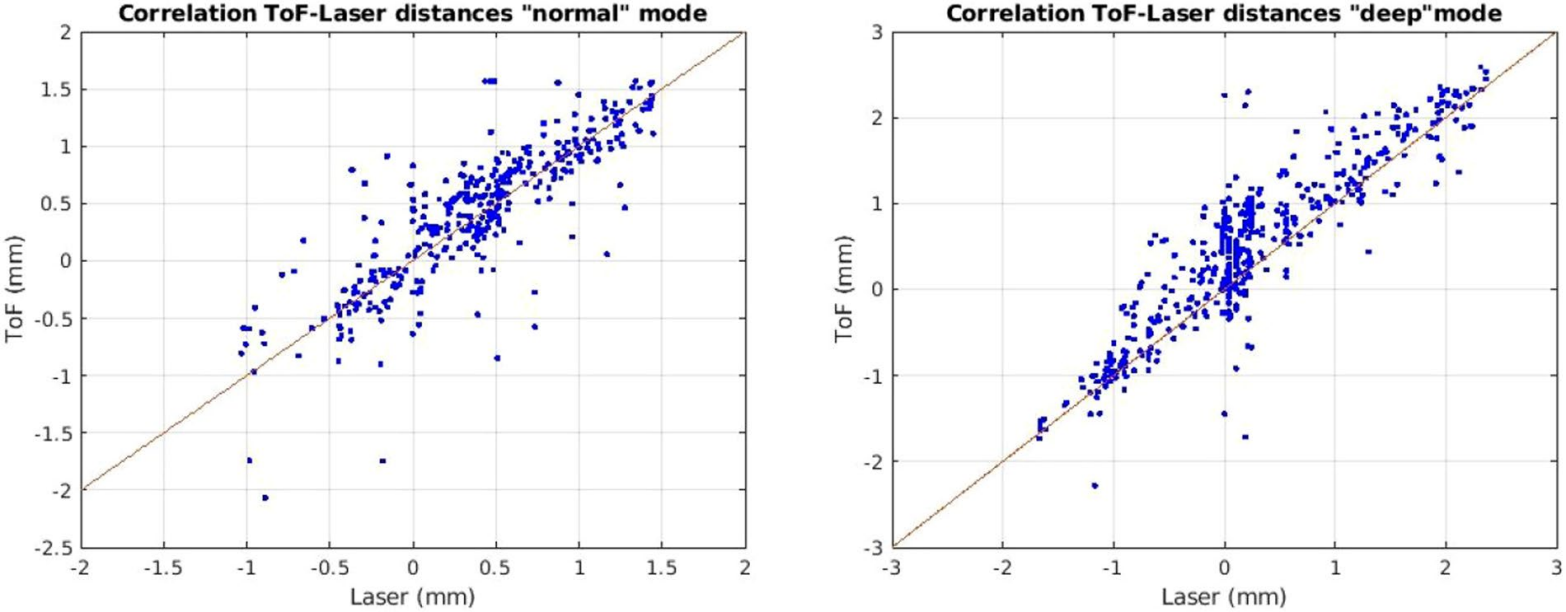
Correlation between laser and ToF distance signal for *normal* (le.) and *deep* (ri.) mode.

In Figure 7 the correlation between the distance signal of the ToF camera and the laser micrometer can be found. As can be seen the overall precision is high. Inaccuracies are due to the low signal-to-noise ratio at low frequencies such as 5 and 10 BPM.

**Fig. 8:**
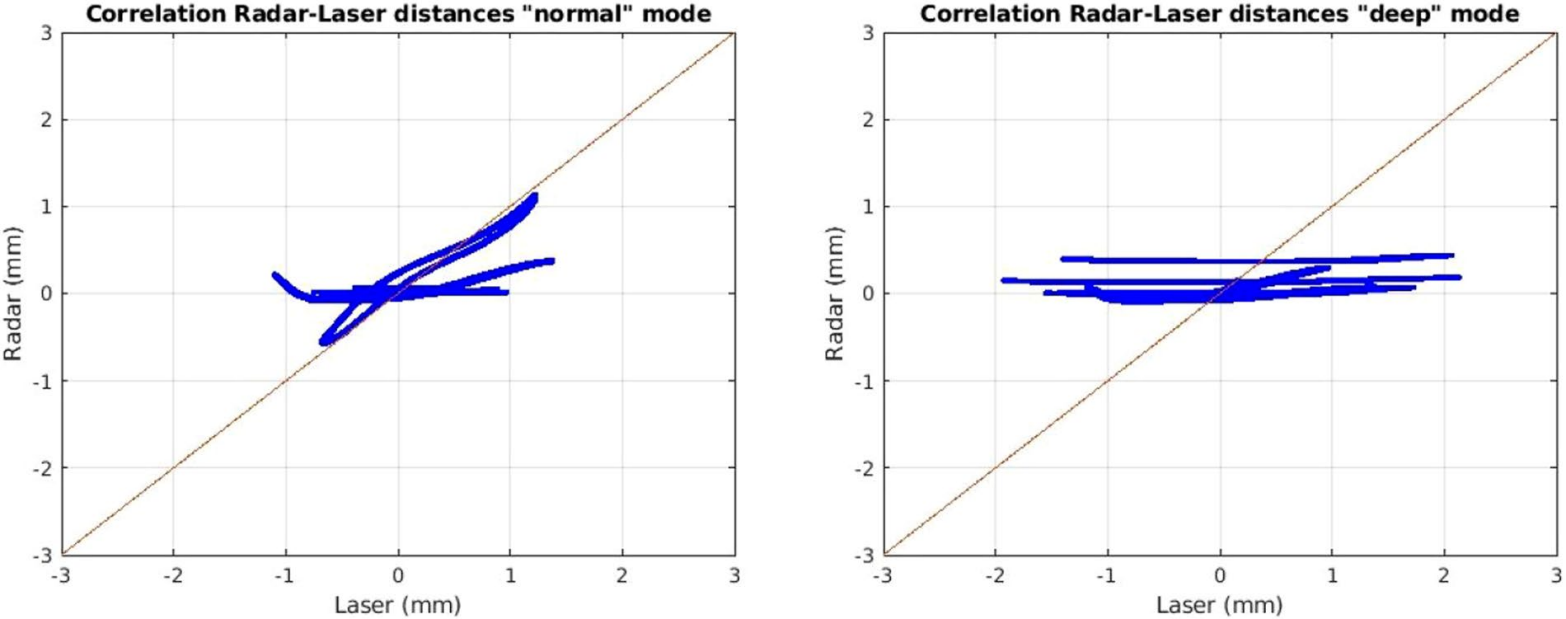
Correlation between laser and radar distance signal for *normal* (le.) and *deep* (ri.) mode.

In Fig. 8 the correlation between the distance signal of the radar sensor and the laser micrometer can be found. The overall precision is high. Imprecisions occur as the Ellipse fitting could not find an ideal ellipse. This leads to a different amplitude for the radar signal from the reference. For further information please refer to the publication by Gleichauf *et al.*^1^. As for us the frequency of the signal is the most important and we showed in Fig. 6 that the frequency is correct and the signals are in sync and in phase, we believe our data is precise enough.

As we showed in our previous publication^1^ the dataset was very helpful for developing non-contact respiratory rate detection algorithms. Nonetheless, it has to be said that the here presented neonatal thorax simulator only covers basic functions as for now. In order to simulate more complex breathing patterns as well as the heartrate further development steps would be required.

## Usage Notes

The csv-files of the laser and radar data can easily be used and read by i.e. Matlab or any editor. The radar IQ values might be needed to be converted to distance values using the formula above (see Eq. 3). For the ToF data the timestamp has to be read from the png filename first. The depth values (z-value) can easily be written into a pointcloud.

**Table 4:** Overview scripts for generating the dataset.

Folder name	Filename	Function
Radar	iqtocsv.sh	Writes raw IQ-values into csv-file.
ToF	pcl_to_png.cpp	Converts ToF pointcloud to png.
Laser	convert.m	Calculates Unix timestamps for laser and writes into csv-file.
	csv_distance_publisher.cpp	Reads data from csv-file and publishes as ROS-topic.

## Data Availability

Our code for generating the dataset is available within the figshare repository within the scripts folder. An overview over the files can be found within Table 4. For using the laser and ToF C++ code ROS (Kinetic or higher version) needs to be installed. For using the Matlab file (convert.m) a Matlab installation is necessary (i.e. version MATLAB R2022a). Furthermore the code for generating the signals simulated by the speaker can be found within the scripts folder, too. Our code may be used under the following terms. The material may not be used for commercial purposes. When using it appropriate credits need to be made. The Creative Commons CC BY-NC 4.0 applies.

## References

[1] Gleichauf, J. et al. Automated non-contact respiratory rate monitoring of neonates based on synchronous evaluation of a 3d time-of-flight camera and a microwave interferometric radar sensor. *Sensors* **21**, 10.3390/s21092959 (2021). 10.3390/s21092959 PMC8122919 33922563

[2] Gleichauf J, Herrmann S, Niebler C, Koelpin A (2024). Figshare.

